# Intravenous infusion of human bone marrow mesenchymal stromal cells promotes functional recovery and neuroplasticity after ischemic stroke in mice

**DOI:** 10.1038/s41598-017-07274-w

**Published:** 2017-07-31

**Authors:** Eliana Sammali, Claudia Alia, Gloria Vegliante, Valentina Colombo, Nadia Giordano, Francesca Pischiutta, Giorgio B. Boncoraglio, Mario Barilani, Lorenza Lazzari, Matteo Caleo, Maria-Grazia De Simoni, Giuseppe Gaipa, Giuseppe Citerio, Elisa R. Zanier

**Affiliations:** 1 Department of Neuroscience, IRCCS – Istituto di Ricerche Farmacologiche Mario Negri, Via La Masa,19, 20156, Milano, Italy; 2 Department of Cerebrovascular Diseases, Fondazione IRCCS – Istituto Neurologico Carlo Besta, Milano, Italy; 3 Neuroscience Institute, CNR, Pisa, Italy; 4 Scuola Normale Superiore, Pisa, Italy; 5 Laboratory for Cell and Gene Therapy “Stefano Verri”, ASST-Monza, San Gerardo Hospital, Monza, Italy; 6 Tettamanti Research Center, Pediatric Department, University of Milano-Bicocca, Monza, Italy; 7 Cell Factory, Unit of Cell Therapy and Cryobiology, Fondazione IRCCS Ca’ Granda Ospedale Maggiore Policlinico, Via F. Sforza 35, 20122 Milano, Italy; 8 School of Medicine and Surgery, University of Milano-Bicocca, Milano, Italy; 9 Neurointensive Care, ASST-Monza, San Gerardo Hospital, Monza, Italy

## Abstract

Transplantation of human bone marrow mesenchymal stromal cells (hBM-MSC) promotes functional recovery after stroke in animal models, but the mechanisms underlying these effects remain incompletely understood. We tested the efficacy of Good Manufacturing Practices (GMP) compliant hBM-MSC, injected intravenously 3.5 hours after injury in mice subjected to transient middle cerebral artery occlusion (tMCAo). We addressed whether hBM-MSC are efficacious and if this efficacy is associated with cortical circuit reorganization using neuroanatomical analysis of GABAergic neurons (parvalbumin; PV-positive cells) and perineuronal nets (PNN), a specialized extracellular matrix structure which acts as an inhibitor of neural plasticity. tMCAo mice receiving hBM-MSC, showed early and lasting improvement of sensorimotor and cognitive functions compared to control tMCAo mice. Furthermore, 5 weeks post-tMCAo, hBM-MSC induced a significant rescue of ipsilateral cortical neurons; an increased proportion of PV-positive neurons in the perilesional cortex, suggesting GABAergic interneurons preservation; and a lower percentage of PV-positive cells surrounded by PNN, indicating an enhanced plastic potential of the perilesional cortex. These results show that hBM-MSC improve functional recovery and stimulate neuroprotection after stroke. Moreover, the downregulation of “plasticity brakes” such as PNN suggests that hBM-MSC treatment stimulates plasticity and formation of new connections in the perilesional cortex.

## Introduction

Stroke is the second cause of death and the leading cause of adult neurological disability worldwide^1–3^. Cerebral ischemic stroke accounts for 87% of all stroke cases. Reperfusion therapies with intravenous thrombolysis^4^ and, more recently, with endovascular mechanical thrombectomy^5^ offer efficacious treatments, however treatment rates extracted from hospital-derived databases range from 3.4 to 9.1% for patients with acute ischemic stroke and the rates of delivery of intra-arterial treatment are far lower^6^. The time window of pharmacological neuroprotection appears to be quite short. However recovery/compensation of neurological function can continue for months after stroke depending on the post-ischemic plasticity milieu and the extent of cortical reorganization^7^. Conventional rehabilitation has been shown to improve functional recovery to some extent^8^. Strategies that can increase and prolong post-ischemic plasticity are urgently needed.

Experimental data show that delivery of mesenchymal stromal cells (MSC) after ischemic stroke reduce toxic events and promote brain restorative processes, with improvements in neurological outcome^9–12^. These results have led to the introduction of MSC-based therapy in pilot clinical trials showing safety^13–16^ and preliminary functional improvement in stroke patients^17^. The European Medicines Agency (EMA) by regulation No. [EC] 1394/2007 of the European Commission^18^ now considers MSC as advanced therapies medicinal products (ATMPs)^19, 20^. However, additional steps are needed in the development of MSC transplantation as a therapy for ischemic stroke^21^. Indeed, further pre-clinical studies are required to understand the mechanisms by which MSC exert their beneficial effects and to maximize their potential benefit. In this process, the use of human bone marrow derived MSC (hBM-MSC) obtained according to Good Manufacturing Practices (GMP), ensuring cell production under the highest standards of aseptic and validated conditions, maximizes the safety and quality of the medicinal product and increases translatability of preclinical results.

MSC are involved in multiple protection and repair mechanisms among which the secretion of neurotrophic factors^22–24^, promotion of angiogenesis^25–27^, neurogenesis and synaptic plasticity^28–30^, and action on immune responses^31–33^. Moreover, MSC are involved in brain remodeling after injury^34, 35^. However, little is known about MSC contribution to cerebral circuit reorganization. Neuronal networks after stroke are impaired not only as a consequence of neuronal death but also because of a direct effect on excitability and synaptic contacts in injured but viable neurons associated to Ca^2+^ overload. The extracellular matrix (ECM) has a central role in the maintenance of microenvironmental homeostasis and neuronal connectivity. Perineuronal nets (PNN) are a specialized form of ECM composed by chondroitin sulfate proteoglycans (CSPGs) that specially surround cell bodies, apical dendrites and the initial axonal segments of some neurons^36–39^. PNN deposition around neurons helps to stabilize the neuronal connections and restricts plastic changes^40–42^. PNN preferentially surround GABAergic interneurons expressing parvalbumin (PV) corresponding to fast-spiking interneurons, which play an important role in the control of neural circuital activity^43^. Here we hypothesized that hBM-MSC treatment would improve stroke recovery by downregulating the molecules that inhibit structural rearrangements, thus promoting the formation of new connections in the perilesional cortex. Aims of the present study are to assess the long-term effects on functional and histopathological outcome of GMP-compliant hBM-MSC in a murine stroke model by right transient middle cerebral artery occlusion (tMCAo), and to understand their effects on neuronal plasticity measured by the expression of PV-positive neurons and PNN.

## Results

### hBM-MSC expansion and characterization

hBM-MSC were expanded until passage 4 (P4), and fold increase and viability were consistently assessed from P0 or from P1 to P3 passages. Four different cell expansions (from four distinct BM sources, named MSC-Bank#1, MSC-Bank#2, MSC-Bank#3, MSC-Bank#6) were performed. Growth rates (expressed as fold increase of cells) were similar at each passage indicating a reproducible kinetics during all phases of cell culture (Table 1). Cell viability was always very high (≥89%) during all the expansion steps. Based on cell availability, transplantation experiments were performed by using hBM-MSC expanded from one single source (MSC-Bank#2) which had a fold increase of 259.0, 267.1, 204.3 at P1, P2, and P3 respectively and a cell viability of 88.4%, 94.1%, 96.6%, and 93.0% at P0, P1, P2 and P3 respectively.

**Table 1:** hBM-MSC growth (fold increase) and viability (%) according to the expansion phase. Fold increase and viability were assessed from P0 to P3 passages.

	P0	P1	P2	P3
Fold increase*	n.a.	176.5 (±41.12)	182.8 (±88.86)	132.5 (±63.98)
Viability*	89.3% (±1.44)	95.4% (±0.68)	96.2% (±1.41)	93.0% (±0.99)

*Values are expressed as mean ± SE of four distinct hBM-MSC expansion experiments obtained from distinct sources.

**Figure 1:**
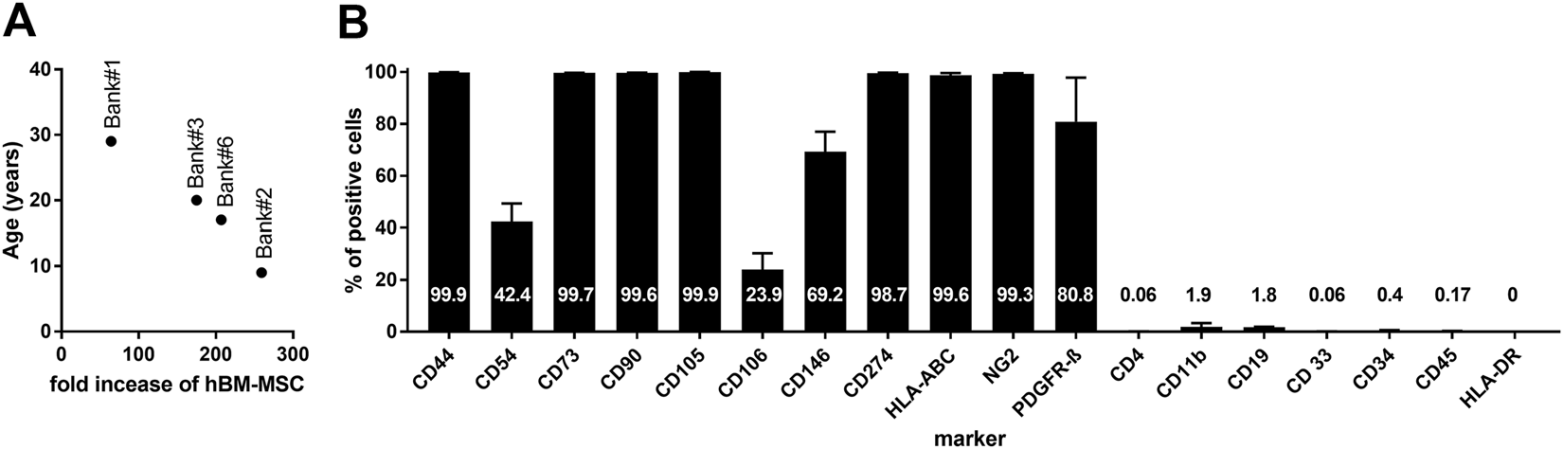
Age related cell kinetics and immunophenotypic characterization of cultured hBM-MSC. (**A**) Correlation between donor age and P0-P1 fold increase from 4 donors (Bank # 1, 3, 6 and 2) shown in descendent order of age. Significant inverse correlation between donor age and kinetic growth curve was found (p = 0.0147, r = −0.985). (**B**) The expression of several immunophenotypic markers was determined by flow cytometry using specific monoclonal antibodies. Marker’s expression is indicated in bars as percentage of positive cells. Markers expected as positive are listed from CD44 to PDGFR-β whereas markers expected as negative are listed from CD4 to HLA-DR. Data are mean + SD from three independent experiments.

In order to assess the impact of donor age on hBM-MSC kinetic expansion we correlated donor age with P0-P1 fold increase according to the criteria used in the context of GMP manufacturing procedures^44, 45^. We found, as expected, an inverse correlation between donor age and kinetic growth curve (p = 0.0147, Fig. 1A).

Immunophenotype was performed at P4 and expression of each marker is reported in Fig. 1B. The phenotypic profile was consistent with the minimum criteria established by the International Society of Stem Cell Research for the characterization of cultured MSC^20^ and was consistent with the immunological profile described in other recent reports^46, 47^.

### hBM-MSC infusion triggers functional sparing in stroke mice

**Figure 2:**
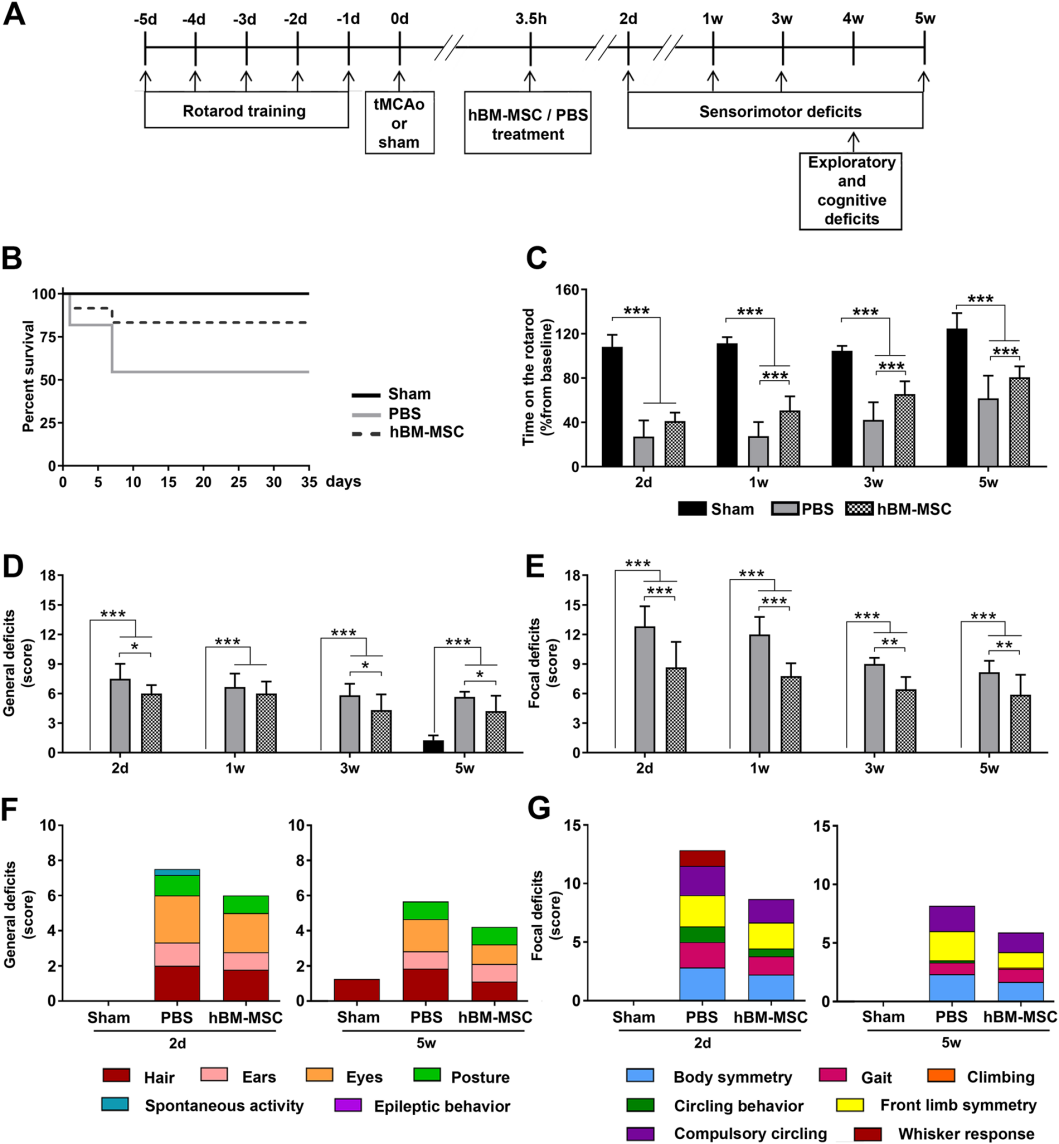
hBM-MSC treatment reduces sensorimotor deficits in stroke mice. (**A**) Experimental design. Initially mice were trained on the accelerated rotarod daily from −5d to −1d to generate stable baseline values. hBM-MSC were injected intravenously (IV) 3.5 h after tMCAo. Sensorimotor deficits were assessed at 2d, 1w, 3w and 5w. Exploratory and cognitive functions were evaluated at 4w measured by the open field and the novel object recognition (NOR) tests. Mice were sacrificed 5w after tMCAo. (**B**) Survivals in sham, tMCAo hBM-MSC and tMCAo PBS mice are shown by Kaplan-Meier curves. (**C**) Latency to fall off the rod was similarly reduced at 2d in both groups of stroke mice. hBM-MSC treatment induced a progressive improvement in the rotarod performance that was significant superior to that of tMCAo PBS mice. (**D**,**E**) The neuroscore revealed an increase in general (**D**) and focal (**E**) deficits in stroke mice as compared to sham. tMCAo hBM-MSC mice showed better general and focal scores compared to tMCAo PBS mice already at 2d, and this difference persisted throughout the observation period. The contribution of each individual parameter on general (**F**) and focal deficits (**G**) in tMCAo hBM-MSC and tMCAo PBS mice for the earliest (2d) and latest (5w) evaluation is shown. Data are mean + SD, n = 6–10, (**B**) Log-rank (Mantel-Cox) test p = 0.06. (**C**) Two-way ANOVA for RM p_treatment_ < 0.0001, p_time_ < 0.0001, p_interaction_ < 0.0001; (**D**) Two-way ANOVA for RM p_treatment_ < 0.0001, p_time_ < 0.0001, p_interaction_ < 0.0001. (**E**) Two-way ANOVA for RM p_treatment_ < 0.0001, p_time_ < 0.0001, p_interaction_ < 0.0001. Tukey’s post-hoc test: *p < 0.05, **p < 0.01, ***p < 0.001.

The *in vivo* experimental protocol is summarized in Fig. 2A. After an initial training in the rotarod, mice underwent either experimental stroke or sham surgery. The lesioned animals (n = 11/12) were randomly assigned to receive an intravenous (IV) infusion of hBM-MSC or phosphate buffered saline (PBS), 3.5 hours (h) after stroke.

Overall mortality after tMCAo was 30.43%. Five out of 11 (45.45%) mice died in the tMCAo PBS group, whereas only 2 out of 12 (16.67%) died in tMCAo hBM-MSC group (Fig. 2B). Difference in mortality between the 2 groups was close to but did not reach significance (Log-rank, Mantel-Cox test, p = 0.06).

Compared to sham mice (n = 6), tMCAo induced significant sensorimotor deficits assessed by both rotarod and composite neuroscore tests at all time points considered (Fig. 2C–G). The rotarod test showed a significant increase in time spent on the rod in tMCAo hBM-MSC compared to tMCAo PBS mice, thus indicating an improved motor coordination and balance (Fig. 2C).

Similarly, composite neuroscore test showed a significant reduction of both general (Fig. 2D) and focal (Fig. 2E) deficits in tMCAo hBM-MSC compared to tMCAo PBS mice. Thus, data show that hBM-MSC not only affect the mouse motor performance and general wellbeing, but also the mouse reactivity, and its response to stimuli^48–52^. The contribution of each individual parameter on general and focal deficits in tMCAo hBM-MSC and tMCAo PBS mice for the earliest (2d) and latest (5w) evaluation is shown in Fig. 2F,G.

**Figure 3:**
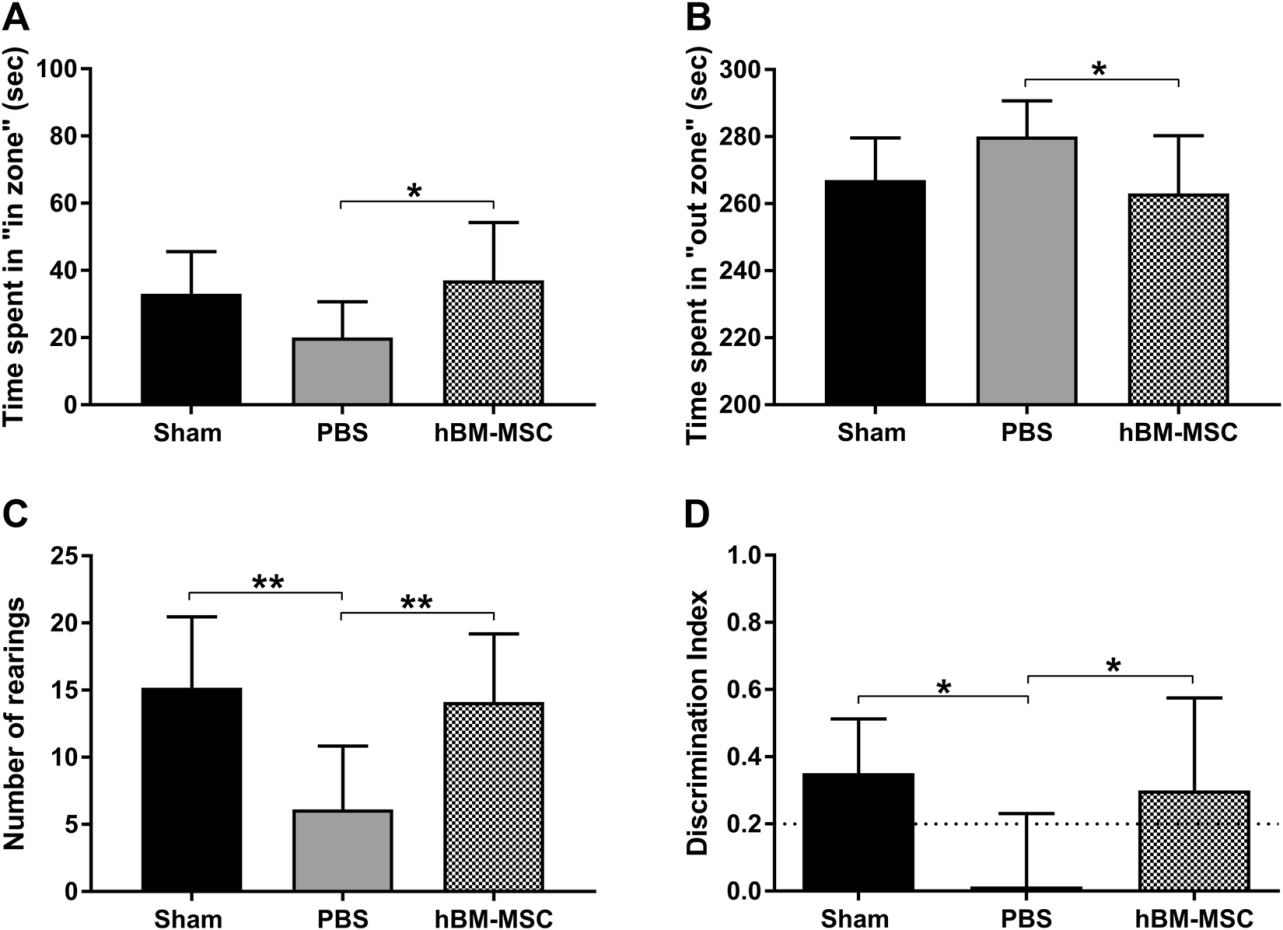
hBM-MSC treatment improves exploratory and cognitive functions in stroke mice. (**A**,**B**) tMCAo hBM-MSC mice spent more time in “in zone” and less time in “out zone” compared to tMCAo PBS mice. (**C**) The number of rearings was significantly increased in tMCAo hBM-MSC compared to tMCAo PBS mice. (**D**) hBM-MSC treatment induced a decrease of cognitive deficits as shown in NOR. The dotted line indicates the limit below which a memory impairment is present. Data are mean + SD, n = 6–10; (**A**) One-way ANOVA p < 0.05; (**B**) One-way ANOVA p < 0.05; (**C**) One-way ANOVA p < 0.01; (**D**) One-way ANOVA p < 0.05. Tukey’s post-hoc test: *p < 0.05, **p<0.01

Anxiety and exploratory behaviors were assessed by the open field test, corresponding to the habituation day of the novel object recognition (NOR) test. Four weeks (w) after stroke, tMCAo hBM-MSC compared to tMCAo PBS mice showed an increase in time spent in the “in zone” (inversely related to anxious behavior, Fig. 3A; p < 0.05) and a decrease in time spent in the “out zone” (directly related to anxious behavior, Fig. 3B; p < 0.05)^53, 54^. Moreover, tMCAo hBM-MSC mice showed a significant increase in the number of rearings compared to tMCAo PBS mice (Fig. 3C; p < 0.01). Data show that hBM-MSC improve both anxiety and exploratory behavior at 4w after stroke in mice.

Recognition memory was assessed by the NOR test (Fig. 3D). During the familiarization day, no difference in time spent on the objects were observed between tMCAo hBM-MSC and tMCAo PBS mice indicating a similar interest towards the objects (data not shown, p = 0.41). During the test day, tMCAo PBS mice showed a lower discrimination index (DI) compared to sham mice (Fig. 3D). tMCAo hBM-MSC mice showed a significant improvement of DI compared to tMCAo PBS mice. Indeed, the performances of tMCAo hBM-MSC mice at 4w after injury were comparable to the sham, unlesioned group (Fig. 3D, sham 0.35 ± 0.16, tMCAo PBS 0.01 ± 0.22, tMCAo hBM-MSC 0.30 ± 0.27; p < 0.05), thus showing an improvement on recognition memory.

### hBM-MSC induce protection of cortical neurons in tMCAo mice

**Figure 4:**
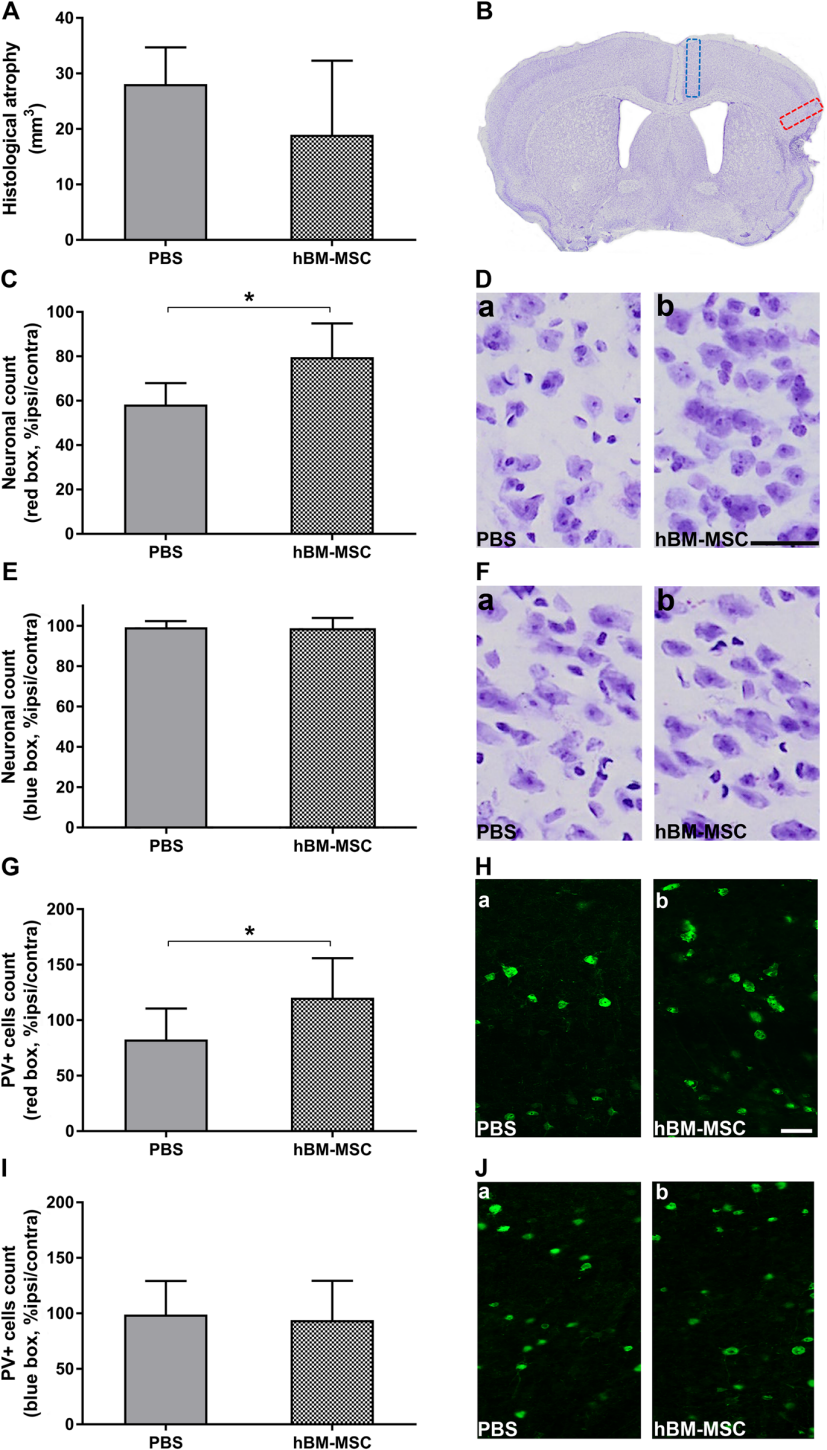
hBM-MSC promote neuronal survival in the perilesional area. (**A**) Stroke induces a similar atrophy in tMCAo hBM-MSC and tMCAo PBS mice 5w post-injury. (**B**) Representative micrograph shows typical brain atrophy and displays the ROI selected for histopathological analyses. Quantification of neuronal death in perilesional cortex (red box) indicates a neuronal rescue after hBM-MSC administration in stroke mice compared to tMCAo PBS mice (**C**,**D**). No difference is observed when a medial ROI (blue box) is selected (**E**,**F**). (**G**) Immunofluorescence analysis in perilesional cortex shows a higher percentage of PV+ neurons in tMCAo hBM-MSC compared to tMCAo PBS mice. (**I**) The percentage of PV+ cells in medial cortex is comparable in tMCAo hBM-MSC and tMCAo PBS mice. (**H**,**J**) Microphotographs show PV stained cells in red box (Ha,b) and in blue box (Ja,b). Data are mean + SD, n = 6–10, unpaired t-test. *p < 0.05. Bar = 50 µm.

Ischemic stroke produced a significant atrophy at 5w in the injured hemisphere compared to the contralateral one. Quantification of brain atrophy showed no significant difference between tMCAo hBM-MSC and tMCAo PBS mice at this time point (Fig. 4A).

In perilesional cortex (red box in Fig. 4B), neuronal count by cresyl violet staining showed a decreased neuronal death in tMCAo hBM-MSC compared to tMCAo  PBS mice (Fig. 4C,D, tMCAo PBS: 58.30 ± 9.64, tMCAo hBM-MSC: 79.66 ± 15.22; p < 0.05). Conversely, considering a different area distant from the lesion site (medial cortex, blue box in Fig. 4B) no difference in neuronal density was observed (Fig. 4E,F; p = 0.88). In addition, no significant differences between groups were detected in striatum, that represents the core of the lesion, (data not shown; tMCAo PBS: 40.48 ± 9.81, tMCAo hBM-MSC: 48.73 ± 12.21; p = 0.23).

The quantification of GABAergic fast-spiking interneurons (parvalbumin; PV-positive cells) similarly showed a greater density of these cells in the perilesional area (red box, Fig. 4B) relative to the homotopic region in the contralateral hemisphere in tMCAo hBM-MSC compared to tMCAo PBS mice (Fig. 4G,H, tMCAo PBS: 82.64 ± 27.93, tMCAo hBM-MSC: 120.2 ± 35.77; p < 0.05). These data are in keeping with the neuronal counts showed in Fig. 4C,D, and indicate that hBM-MSC lead to the neuroprotection of vulnerable GABAergic interneurons.

No difference in the density of PV cells was observed in the medial cortex (blue box, Fig. 4B) distant from the lesion site (Fig. 4I,J; p = 0.79).

**Figure 5:**
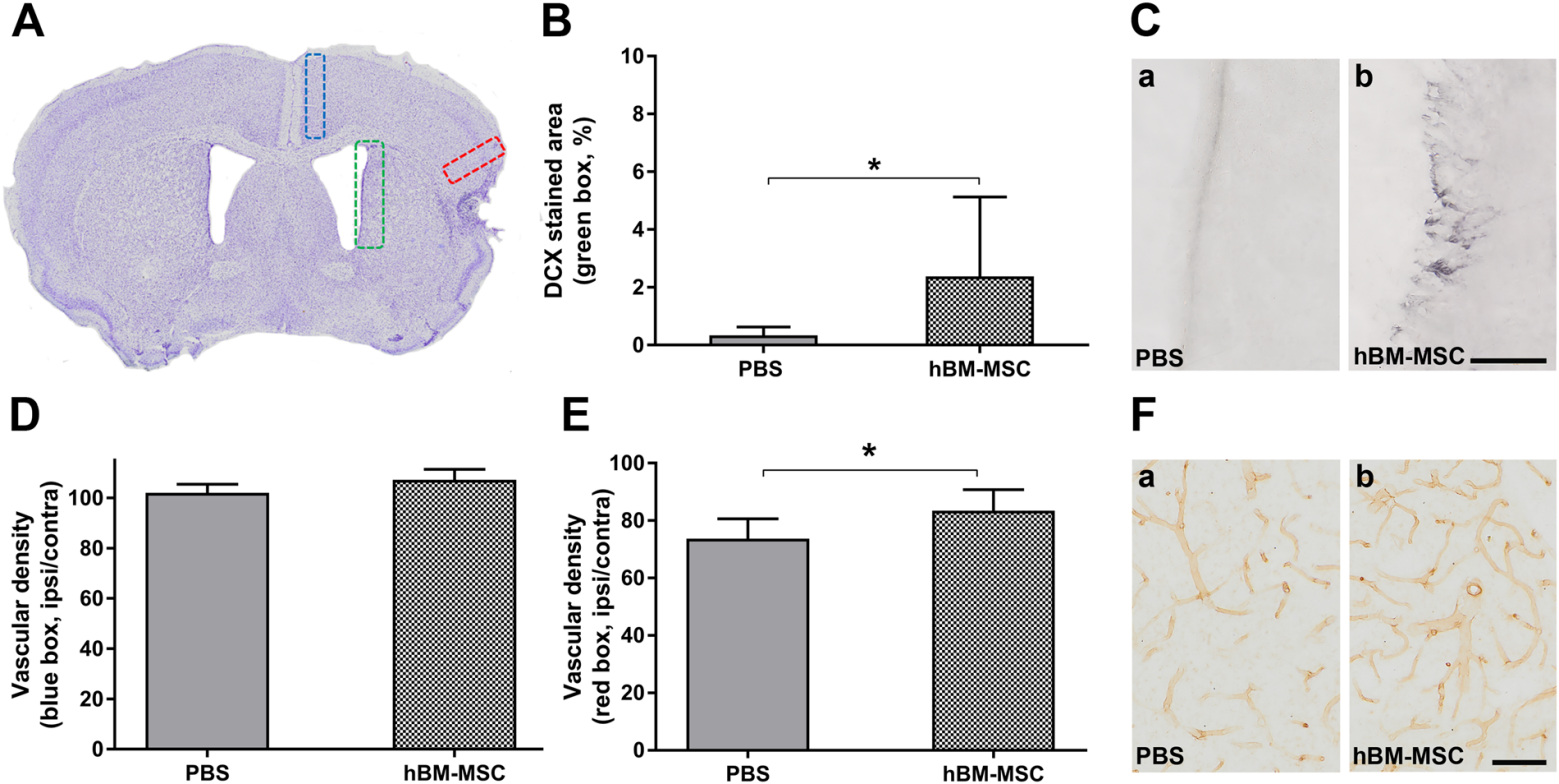
hBM-MSC promote neurogenesis in the subventricular zone and an increase in vessel density. (**A**) Representative micrograph shows the ROI selected for anatomical analyses. Quantification in subventricular zone (green box) indicates a significant increase of DCX in tMCAo hBM-MSC compared to tMCAo PBS mice (**B**,**C**). (**D**) CD31 analysis shows no difference in medial ROI (blue box), however an increase of vessel density is observed in perilesional cortex (red box) of tMCAo hBM-MSC compared to tMCAo PBS mice (**E**,**F**). Data are mean + SD, n = 6–10, unpaired t-test. *p < 0.05. Bar = 50 µm.

Quantification of doublecortin (DCX) staining in subventricular zone (green box, Fig. 5A) showed an increase of newly generated neuroblasts in tMCAo hBM-MSC compared to tMCAo PBS mice (Fig. 5B,C tMCAo PBS 0.34 ± 0.28, tMCAo hBM-MSC 2.38 ± 2.76; p < 0.05). Further, the CD31 immunohistochemical analysis showed no difference in medial region of interest (ROI) (blue box, Fig. 5D; p = 0.18), however an increase of microvessel density was observed in perilesional cortex of tMCAo hBM-MSC compared to tMCAo PBS mice (Fig. 5E,F, tMCAo PBS 73.71 ± 6.88, tMCAo hBM-MSC 83.44 ± 7.37; p < 0.05).

### hBM-MSC administration reduces the density of PV cells surrounded by PNN

**Figure 6:**
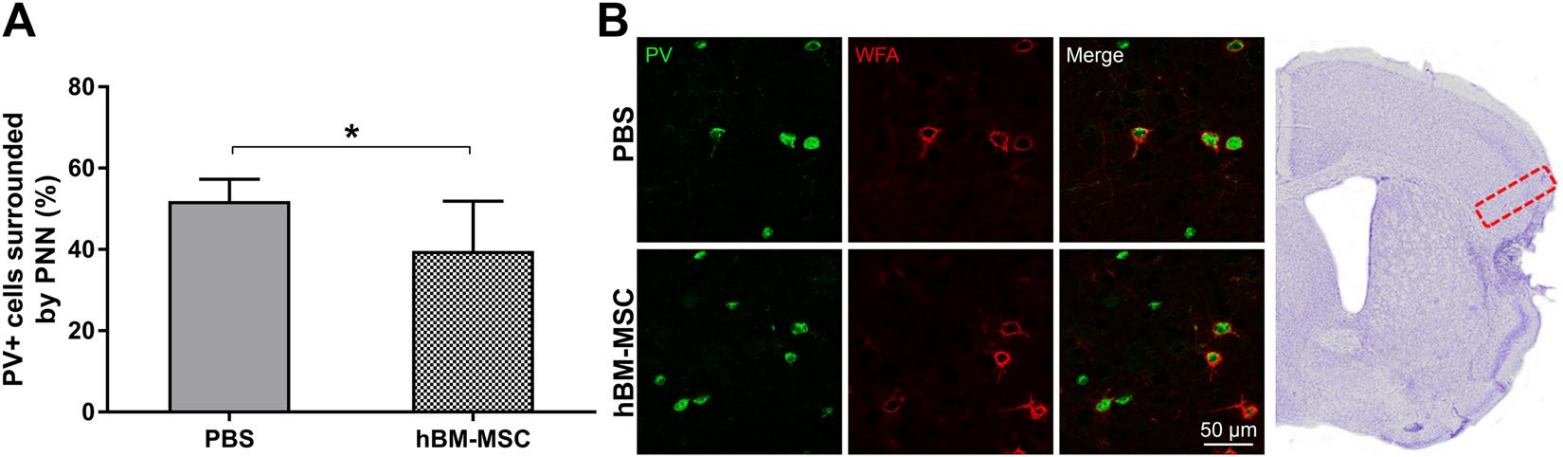
hBM-MSC induce a reduction of PV neurons surrounded by PNN. (**A**) Quantification of PV+ neurons for PNN in perilesional cortex (red box in panel B) 5w post-injury shows lower PV+ cells trapped in PNN after hBM-MSC administration. (**B**) Microphotographs show double immunofluorescence for PV+ cells (green) and PNN (red) in ipsilateral cortex of tMCAo hBM-MSC and tMCAo PBS mice. Data are mean + SD, n = 6–10, unpaired t-test. *p < 0.05. Bar = 50 µm.

PNN mainly surround PV cells and potently limit adult cortical plasticity after injury^41, 42, 55^. Accordingly, we measured the proportion of PV-positive cells surrounded by PNN in tMCAo hBM-MSC and tMCAo PBS mice. This analysis revealed that hBM-MSC transplant reduced the percentage of fast-spiking interneurons positive for PNN in the perilesional tissue (Fig. 6A, tMCAo PBS: 51.93 ± 5.38, tMCAo hBM-MSC: 39.66 ± 12.22; p < 0.05). A reduction of these “plasticity brakes”^40, 56^ suggests that hBM-MSC contribute to cortical circuit reorganization after stroke.

Contralateral cortex showed no changes between the two groups (data not shown; p = 0.55).

## Discussion

We demonstrated that GMP- compliant hBM-MSC improve functional recovery after stroke injury with an early and persistent effect on sensorimotor and cognitive functions up to 5w after stroke. hBM-MSC induce a neuroprotective effect on perilesional cortical neurons in the ipsilateral hemisphere and importantly they promote neural plasticity by reducing PV-positive neurons enwrapped by PNN, thus facilitating brain cortical reorganization.

In the experimental setting, the choice of MSC source and manufacturing protocol is of crucial importance. Autologous, allogeneic and xenogeneic cell transplantation has been widely used in rodent stroke models^9, 57^. Rodent MSC allow syngeneic or allotransplants, which are the conditions faced in the clinical setting. Although autologous transplant minimizes the risk of immune rejection, to isolate and expand MSC requires weeks, thus making such intervention in the acute phase after stroke impossible. Furthermore in support of allogenic transplant for stroke are data showing that aging may influence the therapeutic potential of stem cells^58, 59^ in keeping with our own data showing that age influences hBM-MSC kinetic expansion.

Allogeneic transplant is widely used in the experimental setting and allows acute transplantation with well-established therapeutic effects^9, 60^ and with no significant difference in functional recovery between autologous and allogeneic MSC^61, 62^. The low immunogenic profile of MSC has enabled xenogenic transplantation of human MSC in rodent models of stroke. In the present study, in order to provide direct information on therapeutic translation, we tested hBM-MSC manipulated in GMP conditions^63, 64^, and reproducing all the standards used for clinical studies including storage, transportation and thawing. Culture condition can impact on MSC features and potency^65^, thus MSC produced outside standardized and validated GMP conditions might not possess the biological purity, stability, and activity of cell-based medicinal products ready for clinical use^62^. Here we show the efficacy of GMP manufactured hBM-MSC in a murine model of stroke, providing evidence of their beneficial effects on behavioral and structural outcomes. These results strengthen and expand previous evidences showing protective efficacy of conventionally cultured MSC in preclinical models of stroke^66–70^.

In line with previous observations^25–30^, we show that hBM-MSC induce protective changes on perilesional microvessels and subependimal newly generated neurons, thus confirming that they act through multiple mechanisms. In our study we obtained beneficial effects after systemic infusion of hBM-MSC. We did not investigate the presence of the infused cells in the brain, however we tracked IV infused hMSC in a previous study and showed that hMSC loaded with nanoparticles were detectable in the lungs up to 48 h after surgery, while no cells were found in the brain at 48 h or later time points^71^. Nevertheless infused hMSC produce long-lasting changes as shown by the improvement in anxious behavior, recognition memory performance and sensorimotor function 5w after injury compared to tMCAo PBS mice. These data are consistent with previous studies demonstrating the safety and efficacy of IV administration of MSC in ischemic stroke models^66–70^ and clinical cases^13, 15,72]^ and support the view of MSC-induced therapeutic efficacy through secretion of bioactive factors with neurotrophic/immunomodulatory potential^73–75^.

hBM-MSC can improve the injured host environment by altering the ECM and allowing restorative plasticity through circuit reorganization in the perilesional cortex. By neuroanatomical analysis conducted at 5w after tMCAo we observed a reduced neuronal death in the perilesional area of tMCAo hBM-MSC compared to tMCAo PBS mice. Specifically, we observed a significant protection of fast-spiking GABAergic interneurons, which play an important role in the control of network firing due to their perisomatic synapses onto pyramidal cells^76, 77^. Notably we show that this protection is also associated with a reduction of the percentage of PV-positive interneurons enwrapped by PNN. PNN are considered as “plasticity brakes”, because their reduction promotes network reorganization in the adulthood^78^; in particular, their removal in the perilesional area has been demonstrated to be beneficial for motor function recovery after stroke^41, 79, 80^. Thus, the observation of a decreased percentage of PNN- surrounded interneurons in tMCAo hBM-MSC mice suggests that hMSC treatment may promote network reorganization and functional recovery. Further electrophysiological examinations^81^ are needed to establish the impact of reduced PNN expression on the spiking properties of PV-positive interneurons and network activity.

The mechanisms through which PNN restrict CNS plasticity are still debated. Chondroitin sulfate proteoglycans (CSPGs), that compose PNN may directly inhibit neurite outgrowth by acting as a physical ‘barrier’ that restrains new connections in the mature cortex^82–86^. Recently we showed that the perilesional density of PNN is decreased 30d after photothrombotic stroke in mice^42^ and this is associated with a trend towards spontaneous restoration of motor function. Likewise, a significant degree of spontaneous recovery of function was also detectable in tMCAo PBS mice in the rotarod test one month post-injury. hBM-MSC treatment at this stage spares PV positive cells compared to tMCAo PBS mice and is associated with a further decrease in PNN. Thus, in this case the density of PNN is regulated independently from interneuron survival, in line with a previous report^87^. Our observations are in agreement with data obtained after treatment with chondroitinase ABC – an enzyme that degrades PNN – in models of focal brain trauma^88^ and stroke^41, 80, 89^. In both conditions, acute brain injury *per se* decreased cortical density of PNN, and chondroitinase ABC treatment in injured mice further reduced PNN and produced significant gains in cortical map plasticity and function.

In conclusion our study shows that intravenous administration of GMP- compliant hBM-MSC increase functional recovery through neuroprotective and plasticizing effects that promote neuroplasticity in stroke mice. Data show that hBM-MSC enhance cell survival and downregulate PNN-surrounded neurons after a cerebral infarct likely enabling plasticity in the perilesional cortex.

## Methods

### hBM-MSC isolation

Isolation and expansion of hBM-MSC was performed according to Good Manufacturing Practices (GMP)-compliant procedures previously described^44, 45^. Prior written and informed consent were obtained from donors and the study was approved by the ethics review board of the ASST-Monza Ospedale San Gerardo. We confirm that all methods were performed in accordance with the ethical guidelines of the ASST-Monza Ospedale San Gerardo. BM-derived total nucleated cells were isolated from the wash-outs of sealed bone marrow collection bags and filters. Cells were washed with 20 ml of saline solution (Baxter) and transferred in 50 ml tubes. Three further washing steps were repeated in order to recover the highest number of cells. The tubes were then centrifuged at 680 g for 5 minutes and the cells resupended in 50 ml of saline solution. After centrifugation at 535 g for 8 minutes, cells were then cultured in T175 culture flasks (Greiner Bio-one) at a cell density of 0.5–1.0 × 10^6^ cells/cm^2^ in medium consisting of alpha-MEM (Invitrogen) containing 5% of Platelet lysate (PL). Culture flasks were then horizontally placed in the incubator at 37 °C with 5% CO_2_.

PL was produced from platelet-rich plasma (PRP) supplemented with 200 IU/ml of heparin (Pharmatex, Milan, Italy) and diluted with plasma to a final concentration of 1 × 10^6^ platelets/µl. The platelet suspension is then frozen at −80 °C in horizontally positioned 50 ml tubes (International PBI). Twelve hours after freezing, aliquots of PRP are thawed at 37 °C and centrifuged at 3350 g for 15 minutes. The PL supernatant is collected and centrifuged at 3350 g for 15 minutes and stored at −20 °C in aliquots of 25 ml until use.

hBM-MSC from 4 different healthy donors were expanded and tested for growth rates and cell viability (Fig. 1A). Among these Bank﻿#2 was chosen for *in vivo* studies. Bank#2 donor details are, ethnicity: North African; sex: female; age: 9 years old.

### hBM-MSC expansion

In order to remove non-adherent cells, at day 2–3 of culture, the medium was removed, the cell monolayer gently washed with saline solution, and fresh complete medium was added. Then each flask is horizontally placed again in the incubator. Culture flasks were monitored by inverted microscope for cell growth and the exhausted medium was replaced with fresh complete medium every 2–3 days. Upon reaching 80% of confluence (day+12 /+14), the adherent cells (Passage 0) are washed with saline solution and detached from the surface of the flask with the specific protease (triple select). The cells were then centrifuged and suspended in complete medium. After cell count, cells were suspended at a concentration of 1 × 10^6^ cells/ml in freezing solution (80% human albumin, 10% DMSO and 10% ACD). Cryogenic vials, containing 1 ml each, were placed at −80 °C for 24 hours, and then transferred in liquid nitrogen vapor phase.

Further cell expansions (P1–P4) were performed from cryopreserved vials according to the number of cells required for the experiments. Cryopreserved hBM-MSC were thawed, and resuspended in alpha-MEM medium for cell counting and viability test. Cells were then cultured in multiple-chamber stack (Corning) at a cell density of 120 ± 20 cells/cm^2^ in complete culture medium (alpha-MEM medium supplemented with 5% PL). The culture chambers were horizontally-positioned in the incubator at 37 °C with 5% PL. Cells were monitored twice a week for cell growth by using an inverted microscope, and half of the exhausted medium was regularly replaced with fresh complete medium. Upon reaching 80% of confluence (day+12/+14), the adherent cells were washed with saline solution and detached from the surface of the chamber with the triple select and seeded again until passage 4 (P4). All the expansions were performed in GMP-compliant class A/class B areas to ensure aseptic conditions in a Cell Factory authorized by Agenzia Italiana del Farmaco (AIFA) for the manufacturing of cell-based medicinal products for advanced therapies (Laboratory for Cell and Gene Therapy “Stefano Verri”).

### hBM-MSC characterization

Cell counting and viability was performed in Burker chamber by trypan blue (Sigma-Aldrich) dye exclusion assay.

In order to extensively characterize the immunophenotype of hBM-MSC several immunological markers were included such as CD90 and CD105 (stromal molecules); CD44, CD54, CD106 and CD146 (cell-cell interaction and adhesion); CD4, CD11b, CD14, CD19, CD33, CD45 (hematopoietic markers); CD34 (endothelial); CD73, CD274, HLA-BC and HLA-DR (immune-regulatory and co-stimulatory molecules); NG2 (Neuron-Glial Antigen 2) and the perivascular cell marker with platelet-derived growth factor-receptor beta (PDGF-Rβ). All markers and antibody combinations are reported in Table 2. Briefly 0.4 × 10^6^ cells were stained with fluorochrome conjugated monoclonal antibodies (mAbs) and incubated for 20 minutes at room temperature in the dark. Samples were centrifuged at 1600 rpm for 5 minutes, washed twice with PBS and analyzed immediately, in a FACSCalibur flow cytometer (BD) equipped with CellQuest software. At least 20,000 events were acquired for each sample. Non-viable cells were excluded by physical gating.

**Table 2:** Combinations of fluorochrome-conjugated monoclonal antibodies used for the immununophenotypic characterization of hBM-MSC.

Tube n	Fluorochrome			
	FITC	PE	PerCP	APC
1	unstained	unstained	unstained	unstained
2	CD34 (IQP)	CD90 (eBiosciences)	CD45 (Becton Dickinson)	CD274 (PDL1) (Biolegend)
3	HLA-DR (IQP)	CD105 (eBiosciences)	CD45 (Becton Dickinson)	CD106 (VCAM) (Becton Dickinson)
4	HLA-ABC (Becton Dickinson)	CD33 (Becton Dickinson)	CD45 (Becton Dickinson)	CD44 (eBiosciences)
5	CD14 (IQP)	CD73 (BD Pharmingen)	CD45 (Becton Dickinson)	CD19 (Becton Dickinson)
6	CD146 (eBiosciences)	PDGFRβ (Becton Dickinson)	CD45 (Becton Dickinson)	NG2 (R&D)
7	CD4 (Becton Dickinson)	CD54 (Becton Dickinson)	CD45 (Becton Dickinson)	CD11b (MAC-1) (Becton Dickinson)

### Animals

Male C57BL/6 J mice (9 weeks of age; Harlan Laboratories, Udine, Italy) were housed in a specific pathogen-free vivarium at a constant temperature (21 ± 1 °C) with a 12 h light–dark cycle and *ad libitum* access to food and water. All experiments were conducted in conformity with institutional guidelines that are in compliance with national and international laws and policies (Italian Governing Law: D.lgs 26/2014; Authorization n.19/2008-A issued March 6, 2008 by Ministry of Health; Mario Negri Institutional Regulations and Policies providing internal authorization for persons conducting animal experiments: Quality Management System Certificate – UNI EN ISO 9001:2008 – Reg. No. 6121; the NIH Guide for the Care and Use of Laboratory Animals (2011 edition) and EU directives and guidelines (EEC Council Directive 2010/63/UE). The Statement of Compliance (Assurance) with the Public Health Service Policy on Human Care and Use of Laboratory Animals have been recently reviewed (9/9/2014) and will expire on September 30, 2019 (Animal Welfare Assurance #A5023-01). All experiments followed the ARRIVE guidelines and were approved by the IRCCS-IRFMN Animal Care and Use Committee and by the Italian ‘Istituto Superiore di Sanita’ (code: 32/13D). Mice were randomly allocated for surgery and treatments by a list randomizer (www.random.org/list), taking care to distribute them equally across experimental days. All surgeries were performed by the same investigator. All behavioral and neuroanatomical evaluations were performed by investigators unaware of injury/treatment status of the animals.

### Experimental design

Experiments were planned following the experimental design illustrated in Fig. 2A. Mice were subjected to sham surgery (n = 6) or 60 minutes of tMCAo (n = 11/12) followed by intravenous infusion of hBM-MSC or PBS, 3.5 h after surgery. Sensorimotor deficits were assessed at 2d, and at 1w, 3w and 5w by rotarod and composite neuroscore tests. Exploratory and cognitive functions were evaluated at 4w by NOR test. Mice were sacrificed at 5w for histopathological evaluations. All surgeries were performed by the same investigator, blinded to the experimental groups. All behavioral, histological and biochemical evaluations were done by investigators unaware of injury or treatment status of the animals.

### Surgery

Anesthesia was induced by 3% isoflurane inhalation in an N_2_O/O_2_ (70/30%) mixture and maintained by 1 to 1.5% isoflurane inhalation in an N_2_O/O_2_ (70/30%) mixture. Transient ischemia was achieved by tMCAo by means of a siliconized filament (7-0, Doccol Corporation) introduced into the internal carotid artery and advanced to block the MCA for 60 minutes^71^. Sham-operated mice received identical anesthesia and surgery without artery occlusion.

### hBM-MSC transplantation

For *in vivo* experiments hBM-MSC at P4 were used. Cells were detached and resuspended in PBS. Cell number was evaluated by light microscopy. Viability was evaluated by the trypan blue exclusion test and cell concentration was adjusted to 1 × 10^6^ cells/200 μl PBS. Three hours and 30 minutes after surgery, 1 × 10^6^ hBM-MSC or PBS alone (200 μl) were IV injected through the tail vein.

### Sensorimotor deficits

Sensorimotor deficits were evaluated by rotarod and composite neuroscore tests. Motor coordination and balance was assessed by rotarod test before injury (day 0) and 2d, 1w, 3w and 5w after tMCAo. Before surgery, mice received a training for 5 days. Mice were positioned on the smooth rotating rod, which was then accelerated at a constant rate of 0.12 r.p.m./second from 4 to 40 r.p.m. over 5 minutes. The latency to fall was recorded. For each evaluation, three trials were given to each animal (inter-trial interval: 10 minutes). The average of the three trials was calculated. Data are expressed as percentage of the baseline value. Two consecutive passive rotations without walking, but accompanying the rod, were considered as a fall^90^.

The composite neuroscore was performed at 2d, 1w, 3w and 5w after tMCAo to evaluate the effects of hBM-MSC on the mouse general status and focal neurologic dysfunction as described previously^50–52, 91^. The score ranges from 0 (no deficits) to 56 (representing the poorest performance in all items) and is calculated as the sum of the general and focal deficits (both ranging from 0 to 28). Results are expressed as general deficits, which included the following (scores): hair (0 to 2), ears (0 to 2), eyes (0 to 4), posture (0 to 4), spontaneous activity (0 to 4), and epileptic behavior (0 to 12); and focal deficits: body asymmetry (0 to 4), gait (0 to 4), climbing on a surface inclined at 45° (0 to 4), circling behavior (0 to 4), front limb symmetry (0 to 4), compulsory circling (0 to 4) and whisker response to light touch (0 to 4).

### Exploratory and cognitive deficits

Four weeks after tMCAo, mice were subjected to the NOR test to assess recognition memory performance. Anxiety and exploratory behaviors were assessed by the open field test, corresponding to habituation trial (day one) of the NOR test. A gray Perspex square arena surrounded by walls (40 × 40 × 30 cm) with the floor divided into 25 squares (8 × 8 cm), placed in a specific room separated from the operator’s room was used. The nine central squares (24 × 24 cm) represent the “in zone” and the surrounding border zone the “out zone”. The following objects were used: a black plastic cylinder (4 × 5 cm), a glass vial with a white cup (3 × 6 cm), and a metal cube (3 × 5 cm). The task started with a habituation trial (open field test) during which the animals were placed in the center of the empty arena for 5 minutes, and their movements were recorded by Ethovision XT, 5.0 (Noldus Information Technology, Wageningen, The Netherlands). The day after, mice were again placed in the same arena containing two identical objects (familiarization phase). Exploration was recorded in a 10 minutes’ trial by an investigator blinded to surgery and to treatment. Sniffing, touching, and stretching the head toward the object at a distance of no more than 2 cm were scored as object investigation. Twenty-four hours later (test phase), mice were again placed in the arena containing two objects, one of the objects presented during the familiarization phase (familiar object) and a new different one (novel object), and the time spent exploring the two objects was recorded for 10 minutes. Anxiety and exploratory related behaviors at the open field were assessed by quantifying the time spent in the “in zone” (inversely related to anxiety behavior) and the “out zone” (directly related to anxiety behavior) of the arena, and the number of rearings (directly related to exploratory behavior)^53, 54^.

Recognition memory was assessed during the test phase and expressed as a discrimination index (DI), i.e., (seconds spent on novel −seconds spent on familiar)/(total time spent on objects). Animals with no memory impairment spent a longer time investigating the novel object, giving a higher DI^71, 92^.

### Tissue processing for histopathological analysis

Five weeks after tMCAo, mice were deeply anaesthetized with ketamine 30 mg/medetomidine 0.3 mg and transcardially perfused with 20 ml of PBS, 0.1 mol/l, pH 7.4, followed by 50 ml of chilled paraformaldehyde (4%) in PBS. The brains were carefully removed from the skull and post-fixed for 6 h at 4 °C, and then transferred to 30% sucrose in 0.1 mol/l PBS for 24 h until equilibration. The brains were frozen by immersion in isopentane at −45 °C for 3 minutes before being sealed into vials and stored at −80 °C until use. Forty µm thick serial sections were cut using a cryostat /sliding microtome (Leica, Germany) from bregma 1.6 to bregma −3.5.

### Anatomical damage

Post-stroke atrophy was calculated on 40 µm coronal brain cryosections stained with cresyl violet as previously described^90^.

An Olympus BX61 microscope, inter-faced with VS-ASW-FL software (Olympus Tokyo, Japan) was used to acquire the all sections at 2x of magnification. Images were acquired on a computer using the image analyzer Analytical Image System (Imaging Research Inc, Brock University, St Catharines, Ontario, Canada), and atrophy was calculated.

### Histopathological analysis

Histological analysis was performed on three coronal sections per mouse at +0.6, 0 and −0.6 mm from bregma, in a 200 µm wide cortical column drawn at the lateral (red box, Fig. 4B), medial (blue box, Fig. 4B) edge of the ischemic tissue. For DCX staining, we analyzed a 200 µm wide column traced along the subependymal zone (green box, Fig. 5A) on two coronal sections per mouse at 0 and −0.4 mm from bregma^27, 75^.

#### Neuronal count

Cresyl Violet stained brain sections were used for neuronal count. An Olympus BX-61-VS microscope, inter-faced with VS-ASW-FL software was used to acquire the whole sections at 20x of magnification. For each mouse, whole striatum and the perilesional area (red box in Fig. 4B) of cortex was analyzed over the ipsilateral and the contralateral hemispheres. The degree of neuronal loss was calculated by pooling the number of stained neurons in the sections of each hemisphere and was expressed as percentage of neurons in the ipsilateral over the contralateral hemisphere normalized over the acquisition area.

Images were analyzed using the open source platform software Fiji (http://fiji.sc/Fiji)^93^ and segmentation was used to discriminate neurons from glia on the basis of cell size^94, 95^.

#### Neuronal protection and plasticity

Immunofluorescence analysis was performed on 40 μm thick coronal sections. Free floating sections were incubated with the anti-parvalbumin antibody (1:300, Synaptic Systems, Germany) and the PNN marker Wisteria floribunda agglutinin (WFA; 1:100, Sigma, USA). The number of PV-positive neurons and PNN was analyzed using a fluorescence microscope (Zeiss, Germany) with a 10x objective, counting in perilesional, lateral cortex area (red box in Fig. 4B) and in the medial cortex (blue box in Fig. 4B) both in the ipsi and contralateral hemispheres by Stereo Investigator software (MBF Bioscience, USA).

#### Neurogenesis and vessel density

Immunohistochemistry was performed on 40 μm thick brain coronal sections using anti-DCX (1:200; Santa Cruz Biotechnology, Santa Cruz, CA, USA) and anti-CD31 (1:100; BD) to measure neurogenesis and vessel density respectively. Positive cells were stained by reaction with 3,3′-diaminobenzidine (Vector Laboratories, Burlingame, CA, USA) as previously described^33, 75^. For each reaction, adequate negative controls were performed. DCX and CD31 stainings were acquired at 20x of magnification by Olympus BX-61-VS microscope. Images were analyzed using software Fiji.The newly generated neuroblasts were quantified as percentage of DCX stained area (green box, Fig. 5A). The vessel density was quantified by overlaying digitalized images with a grid (10 × 10 μm per single square). The vascular network was quantified in lateral (red box, Fig. 4B) and medial (blue box, Fig. 4B) cortex, by counting the number of vessels crossing the grid and normalizing the values over the area analyzed. Data are expressed as the percentage of vessel density in the ipsilateral over contralateral hemisphere.

### Statistical analysis

All the data are represented as mean ± SD. Kaplan Mayer curve of mortality was analyzed by Log-rank (Mantel-Cox) test. Rotarod and composite neuroscore tests were analyzed using a two-way analysis of variance (ANOVA) for repeated measures (RM), followed by Tukey post-hoc test. The open field and NOR tests were analyzed by a one-way ANOVA, followed by Tukey post-hoc test. Histopathological data were analyzed using the unpaired t-test. All statistical analyses were performed using the GraphPad Prism version 6.00 (Graph-Pad Software, San Diego, CA, USA). p-values < 0.05 were considered statistically significant.
